# Empirically Determining Binge/Purge Frequency Thresholds for Differentiating Anorexia Nervosa‐Restricting Subtype vs. Binge–Purge Subtype

**DOI:** 10.1002/eat.24391

**Published:** 2025-02-06

**Authors:** Sophie R. Abber, Devon Peterkin, Carina S. Brown, Thomas E. Joiner, Christina E. Wierenga, Lauren N. Forrest

**Affiliations:** ^1^ Department of Psychology Florida State University Tallahassee USA; ^2^ Eating Disorders Center for Treatment and Research, Department of Psychiatry University of California, San Diego Health California USA; ^3^ Department of Psychology University of Oregon Oregon USA; ^4^ San Diego State University/University of California san Diego Joint Doctoral Program in Clinical Psychology California USA

**Keywords:** anorexia nervosa, binge eating, classification, purging, subtype

## Abstract

**Objective:**

While research supports differentiating anorexia nervosa into binge–purge (AN‐BP) vs. restricting (AN‐R) subtypes, *DSM‐5‐TR* does not provide a specific threshold of binge and/or purge episodes that constitutes an AN‐BP vs. AN‐R diagnosis. Our review of the literature suggests that cutoffs used for defining AN subtypes are rarely reported and, when reported, are highly heterogeneous. Inconsistent subtyping protocols limit generalizability and understanding of AN‐R and AN‐BP differences.

**Method:**

The present study used structural equation modeling (SEM) trees to empirically determine the frequency of binge eating and/or purging that best differentiates AN subtypes. We then compared empirically determined groups on characteristics frequently found to differ between subtypes. Participants were 731 adolescents and adults with AN (94% female, M_age_ = 20, 72% clinically diagnosed with AN‐R) in a partial hospitalization program who completed assessments of AN and comorbid symptoms at intake.

**Results:**

SEM tree analyses yielded four subgroups: past‐month binge/purge frequency 0 (AN‐R; *n* = 396); frequency 1–3 (AN‐BP1; *n* = 101); frequency 4–15 (AN‐BP2; *n* = 130); and frequency > 16 (AN‐BP3; *n* = 98). AN‐R differed from higher frequency groups on 14/22 clinical characteristics, AN‐BP1 differed from higher frequency groups on 11/22 clinical characteristics, and AN‐BP2 differed from higher frequency groups on 2/22 clinical characteristics.

**Conclusions:**

Findings suggest that one binge eating and/or purge episode in the past month provides adequate distinction between subtypes. These findings indicate that the DSM's definition of AN‐BP may need to be revised to specify that the presence of any binge eating or purging, rather than “recurrent” binge eating or purging, is sufficient for subtyping AN.

**Summary**

- Research supports classifying anorexia nervosa into restricting (AN‐R) and binge–purge (AN‐BP) subtypes.
- AN‐BP is characterized by recurrent engagement in binge eating and/or purging, but the *DSM‐5*‐*TR* does not specify a particular threshold for distinguishing between subtypes.
- Variations in how AN subtype is determined may limit research progress.
- Using machine learning to identify an appropriate threshold, we found one binge eating and/or purging episode in the past month adequately distinguishes subtypes.

## 1 Introduction

*DSM‐5‐TR* includes two subtypes of anorexia nervosa (AN): a restricting subtype (AN‐R), where low weight is achieved “primarily through dieting, fasting, and/or excessive exercise,” and a binge/purge subtype (AN‐BP), where “*recurrent episodes* of binge eating or purging behavior” are also present (emphasis added; American Psychiatric Association 2022). Research has generally supported differentiating AN into these subtypes. Compared to those with AN‐R, those with AN‐BP often experience increased cognitive eating disorder (ED) symptoms (Reas and Rø 2018), psychiatric comorbidity (Casper et al. 1980; Garner, Garner, and Rosen 1993; Laessle et al. 1989), suicidality (Bulik et al. 2008; Forcano et al. 2011), impulsivity (Vitousek and Manke 1994; Waxman 2009), punishment sensitivity (Glashouwer et al. 2014), and impairment (Miranda‐Olivos et al. 2021; Mond et al. 2005). However, *DSM‐5‐TR* does not define what constitutes “recurrent” binge eating or purging, leaving the distinction between AN subtypes ambiguous. While this ambiguity has been noted since *DSM‐III‐TR* (Eddy et al. 2002; Garner, Garner, and Rosen 1993; Walsh and Kahn 1997), no research to our knowledge has established a cutoff of binge‐eating and/or purging episodes for differentiating subtypes.

As a result of this ambiguity, research and clinical sites have developed varying definitions of AN‐R and AN‐BP. For example, one clinician may classify a patient with one binge episode and one vomiting episode in the past 3 months as AN‐BP, while another might classify the same patient as AN‐R. Even structured clinical interviews used to diagnose AN, such as the structured clinical interview for DSM‐5 (SCID‐5; (First et al. 2015)) do not operationalize “recurrent” binge eating or purging. Our review of the literature suggests that specific cutoffs used for a diagnosis of AN‐R versus AN‐BP are rarely reported in the methods section of published work. When methods do report definitions of AN‐R and AN‐BP, the cutoffs being implemented vary widely. For example, AN‐BP has been defined as ≥ 3 binge eating and/or purging episodes per month over the past 3 months (Breithaupt et al. (2022)), binge eating and/or purging occurring at least weekly over the past 2 months (≥ 4 episodes per month; Eddy et al. (2002)), binge eating and/or purging at least monthly (≥ 1 episode per month; Garner, Garner, and Rosen (1993)), purging at least once in the past 4 weeks (≥ 1 episode per month; Mond et al. (2005)), or any lifetime purging (Bulik et al. (2008)). Some studies have not found differences between AN subtypes (e.g., Eddy et al. 2002). Such conflicting findings could in part be a result of inconsistent diagnostic protocols. Taken together, the DSM‐5‐TR's vague definition for differentiating AN‐R from AN‐BP limits reliability and generalizability in both clinical and research settings.

While AN‐R and AN‐BP appear to be valid subtypes, the ideal threshold of binge/purge behaviors for distinguishing between subtypes is unclear. An ideal method for determining and validating a specific frequency threshold to differentiate AN‐R and AN‐BP may be structural equation modeling (SEM) trees. SEM trees are a form of exploratory data mining, where models take a continuous construct (e.g., binge eating and purging frequency) and identify specific levels of that construct that best differentiate groups (Brandmaier et al. 2013). SEM trees have been used in ED research to empirically determine severity specifiers for AN (Billman Miller et al. 2024), BED (Forrest, Jacobucci, and Grilo 2022), and OSFED (Ortiz et al. 2021). These SEM tree‐determined severity specifiers have outperformed the *DSM‐5‐TR*'s existing severity specification schemes for AN and BED (Billman Miller et al. 2024; Forrest, Jacobucci, and Grilo 2022) and may thus be a useful approach to assist with accurately and uniformly differentiating AN subtypes.

The present study aimed to empirically determine cutoffs for the frequency of binge eating and/or purging behaviors necessary for an AN‐BP diagnosis. Specifically, we aimed to: (1) determine the precise levels of binge eating and/or purging behaviors that differentiate AN subtypes and (2) compare resulting groups on measures frequently found to differ between AN‐R and AN‐BP. Given that an SEM tree approach might result in multiple binge/purge subgroups, an exploratory aim was to test whether more than one binge/purge subgroup was clinically useful. We did not have specific hypotheses for the exact level of binge‐eating and/or purging behaviors that would differentiate AN subtypes. Based on prior work, we hypothesized that psychiatric comorbidity, suicidality, ED symptoms, punishment sensitivity, impulsivity, and impairment would be higher in the resulting SEM Tree groups defined by higher binge‐eating and/or purging frequency relative to groups defined by lower binge‐eating and/or purging frequency.

## 2 Method

### 2.1 Participants

Participants included 731 adolescents and adults with AN and AN in partial remission enrolled in a partial hospitalization program for EDs (93.8% female; M_age_ = 19.56, SD_age_ = 7.34, range = 12–60 years; 80.4% White, 5.3% Asian, 0.5% Black, 0.1% Native Hawaiian/Pacific Islander; 0.5% Native American/Alaska Native, and 12.3% Other; 15.5% Hispanic/Latino/a; M_BMI_ = 17.91 kg/m^2^, SD_BMI_ = 1.97 kg/m^2^; M_Years of Education_ = 12, SD_Years of Education_ = 3). Participants were recruited as part of a larger study examining naturalistic outcomes of a partial hospitalization program for EDs. All participants met diagnostic criteria for AN and standard admission eligibility criteria consistent with the American Psychiatric Association's guidelines for ED treatment at a higher level of care (Yager et al. 2014). See Brown et al. (2018) and Reilly et al. (2020) for details regarding treatment in the program for adolescents and adults, respectively. Briefly, treatment was based on dialectical behavior therapy (DBT) and family‐based treatment (FBT) and comprised of supervised meals and snacks, individual therapy, family therapy, group therapy, nutritional counseling, psychiatric care, and medical management. At admission, most patients received 10 h of treatment per day, 6 days per week, and were gradually stepped down to 6‐h/day PHP and then intensive outpatient (IOP) as patients made progress in treatment.

### 2.2 Procedure

Patients were offered the option of participating in the study upon admission to the program. Those interested were consented and then completed measures within 14 days (M (SD) = 3.86 (3.92)) postadmission. Informed consent or parental consent plus assent was obtained prior to the initiation of study procedures, and all procedures were approved by the university institutional review board (#180055).

### 2.3 Measures

#### 2.3.1 Diagnostic Interviews

AN diagnosis was established using semi‐structured interviews administered by trained bachelor's‐level research assistants and doctoral‐level trainees. Adults completed either the structured clinical interview for *DSM‐5* (SCID‐5; (First et al. 2015)) or the MINI Neuropsychiatric Interview 7.0 (Sheehan et al. 1998), while adolescents completed either the Schedule for Affective Disorders and Schizophrenia for School‐Age Children (KSADS; Kaufman et al. 1997) or the MINI International Neuropsychiatric Interview for Children and Adolescents (MINI‐KID; (Sheehan et al. 2010)). Assessors were extensively trained in diagnostic interviews and received supervision from two licensed clinical psychologists with expertise in diagnostic interviewing. Assessors attended weekly diagnostic consensus meetings and individual assessment consultations as needed.

#### 2.3.2 Latent Outcome Model

SEM trees are estimated in two steps, the first of which is defining an outcome model. Items from the Eating Disorder Examination Questionnaire (EDE‐Q; (Fairburn and Beglin 2008)) were used as indicators for the latent outcome model of AN diagnosis. The latent variable included three variables that map onto *DSM‐5‐TR* AN criteria: the EDE‐Q restraint subscale (α = 0.88 in our sample) represented criterion A (significant restriction of food intake), item 10 (“Have you had a definite fear that you might gain weight?”) represented criterion B (fear of weight gain), and a composite of items 11 (“Have you felt fat?”), 22 (“Has your weight influenced how you think about (judge) yourself as a person?”), and 23 (“Has your shape influenced how you think about (judge) yourself as a person?”; α = 0.89 in our sample) represented criterion C (feeling fat or overvaluation of shape and weight). All of these EDE‐Q items are rated on a 7‐point (0 to 6) Likert scale, with higher scores indicating greater ED symptoms.

While it would have been ideal to also consider physiological measures as part of the outcome model, we are limited to including the variables available in this dataset. Further, we did not include BMI as an indicator of AN diagnosis for two reasons: (1) BMI has limited utility in signaling AN severity (Billman Miller et al. 2024) and (2) patients entering PHP (i.e., the time point at which data for the present study were collected) have clinically significant AN psychopathology but may have partially or fully weight‐restored at higher levels of care prior to entering the PHP.

#### 2.3.3 Covariates

The second step of SEM Tree estimation is specifying covariates, which split the data into subgroups that significantly differ on the outcome model. Our model included one covariate, which was the sum of binge‐eating frequency and purging frequency in the past month. Binge‐eating frequency (i.e., objective binge episodes) was measured by item 14 on the EDE‐Q: “On how many of these times did you have a sense of having lost control over your eating (at the time you were eating)?”, which is a follow‐up question to item 13 (“In the past 28 days, how many times have you eaten what other people would regard as an unusually large amount of food (given the circumstances)?”). Purging frequency was measured as the sum of EDE‐Q items 16 (“Over the past 28 days, how many times have you made yourself sick (vomit) as a means of controlling your shape or weight?”) and 17 (“Over the past 28 days, how many times have you taken laxatives as a means of controlling your shape or weight?”). Binge‐eating and purging frequency were each capped at 56 (i.e., twice daily in the past month) to account for extreme outliers.

#### 2.3.4 Validation Measures

After reviewing the literature on constructs that may differ between AN subtypes, we selected all measures from the Clinic's assessment battery that capture these constructs. Our intention was to be as broad as possible, given that we were less concerned with the specific measures on which subgroups did or did not differ and instead wanted to assess the overall pattern and number of similarities or differences among groups. When there were two available measures for a construct, we included both measures to gain more confidence in whether the pattern of results was general for certain constructs (e.g., ED pathology) or specific to particular measures of constructs (e.g., BIS/BAS vs. SPSRQ).

##### 2.3.4.1 ED Symptoms

The 28‐item (Fairburn and Beglin 2008) and the 45‐item Eating Pathology Symptoms Inventory (EPSI; (Forbush et al. 2013)) measured overall ED symptoms. With respect to the EDE‐Q, we included the dietary restraint, eating concerns, shape concerns, and weight concerns subscales as well as the Global score. The Global score and subscale scores had good internal consistency (α = 0.77–0.94).

We included the EPSI subscales as additional indicators of ED symptoms, to ensure that our latent outcome model (which is modeled from EDE‐Q items) and subsequent comparisons of groups defined by the outcome model (including on EDE‐Q subscales) were not due to the fact that groups were defined and compared on items/constructs stemming from a single measure. We used the EPSI body dissatisfaction, binge‐eating, cognitive restraint, purging, restricting, and excessive exercise subscales, which were determined to be the most theoretically relevant to AN symptoms for the purpose of the current study. Each subscale had at least adequate internal consistency (α = 0.72–0.93).

##### 2.3.4.2 Psychiatric Comorbidity

Current psychiatric comorbidities, including mood disorders, anxiety disorders (including panic disorder, agoraphobia, social anxiety disorder, specific phobia, and generalized anxiety disorder), alcohol use disorder, and substance use disorders, were assessed using the clinical interviews described above. We selected these diagnoses given past work suggesting AN subtypes differ on these comorbidities (Casper et al. 1980; Garner, Garner, and Rosen 1993; Laessle et al. 1989).

##### 2.3.4.3 Suicidality

For participants who completed the MINI, whether or not they had a past suicide attempt was assessed. Additionally, we used the five yes/no suicidality component items of the MINI to quantify suicide risk into low, moderate, and high categories.

##### 2.3.4.4 Impulsivity

The 40‐item novelty‐seeking subscale of the Temperament and Character Inventory (TCI‐NS) (Cloninger et al. 1994) was used to measure impulsivity. The TCI‐NS showed adequate internal consistency (*α* = 0.71).

##### 2.3.4.5 Punishment Sensitivity

We included two indices of punishment sensitivity: the Sensitivity to Punishment subscale of the 24‐item shortened Sensitivity to Punishment/Sensitivity to Reward Questionnaire (SPSRQ; (Cooper and Gomez 2008)) and the 24‐item Behavioral Inhibition System, part of the Behavioral Inhibition System/Behavioral Activation System (BIS/BAS; (Carver and White 1994)). The SPSRQ‐SP (*α* = 0.82) and BIS (*α* = 0.73) showed adequate internal consistency. We included both measures to assess sensitivity to general punishment cues (as in the BIS/BAS, e.g., “making mistakes”) and specific punishment cues (as in the SPSRQ, e.g., “risky jobs”).

##### 2.3.4.6 Impairment

The 16‐item Clinical Impairment Assessment (CIA; (Bohn and Fairburn 2008)) measured ED‐related impairment. The CIA showed excellent internal consistency (*α* = 0.95).

##### 2.3.4.7 Clinical Diagnosis

We calculated the percentage of individuals in each SEM tree group who received an AN‐BP clinical diagnosis, based on the Clinic's threshold of ≥ 12 binge‐eating and/or purging episodes in the past 3 months.

### 2.4 Statistical Analysis

Analyses were conducted in SPSS version 28.0 and in R using the packages foreign (Team et al. 2020), haven (Wickham, Miller, and Smith 2023), openMx (Neale et al. 2016), and lavaan (Rosseel 2012). Code for the SEM trees is available as Supporting Information.

#### 2.4.1 Normality, Assumptions, and Missing Data

Data were examined and conformed to assumptions of normality. We used Levene's test to test the equal variances assumption, and Welch‐corrected ANOVAs and contrasts are reported when indicated.

Listwise deletion was used to handle missingness on binge and purge frequencies for SEM trees, given that binge or purge data were missing for only *n* = 16 (2%). Full information maximum likelihood to handle missingness for SEM trees and forests and pairwise deletion was used to handle missingness for comparisons among SEM Tree subgroups.

##### 2.4.1.1 SEM Trees

**FIGURE 1:**
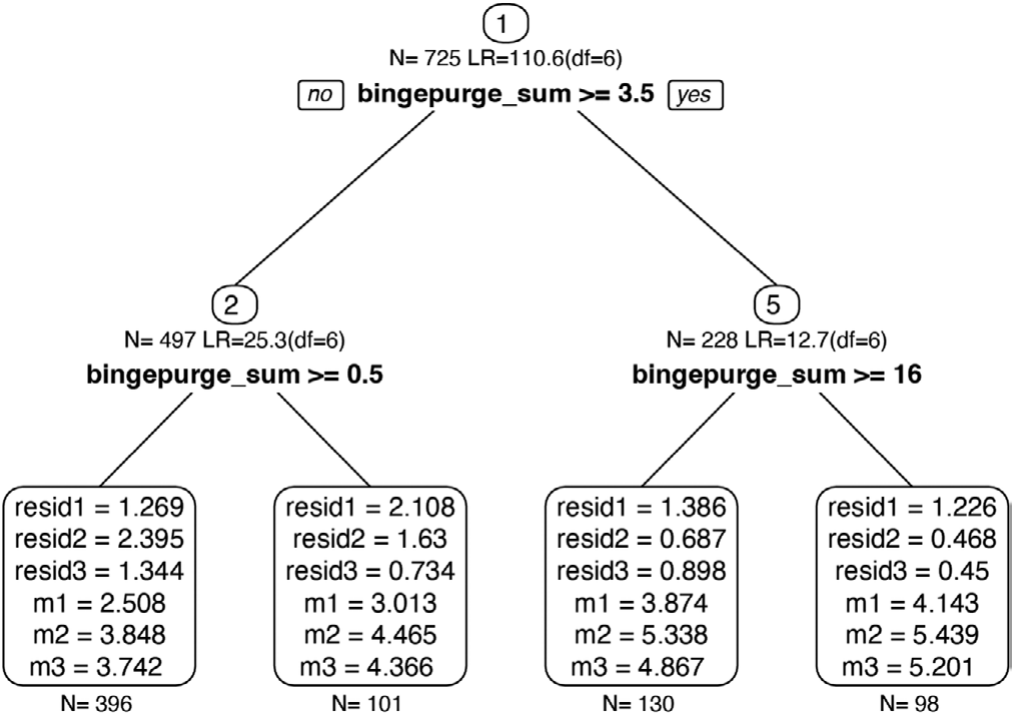
Structural equation model tree results identifying the frequency of past‐month binge eating and purging that differentiates anorexia nervosa subtypes. LR = likelihood ratio; resid1 = residual variance of *DSM‐5‐TR* AN criterion A (EDE‐Q restraint subscale); resid2 = residual variance of *DSM‐5‐TR* AN criterion B (EDE‐Q fear of weight gain item), resid3 = residual variance of *DSM‐5‐TR* AN criterion C (composite of EDE‐Q feeling fat, overvaluation of shape, and overvaluation of weight items). m1 = manifest mean of *DSM‐5‐TR* AN criterion A, m2 = manifest mean of *DSM‐5‐TR* AN criterion B, m3 = manifest mean of *DSM‐5‐TR* AN criterion C.

Since only three indicators were used for the latent AN diagnosis outcome model, model fit could not be assessed. The SEM tree recursively separated data into subgroups that explained the most variance in the latent AN diagnosis outcome model. Resulting subgroups were based on values (i.e., splits) of the covariates. A ‘fair’ splitting criterion (Brandmaier et al. 2013), where the sample is randomly divided into two equal parts, was used. The latent outcome model is first compared at every possible value of the covariate. Then, the value resulting in the largest model fit improvement is selected and evaluated in the other half of the sample. A retained split indicates that a particular value of binge/purge frequency differentiates subgroups on the outcome model (see Figure 1).

#### 2.4.2 Planned Comparisons

ANOVAs with planned Helmert contrasts were used to compare each binge/purge frequency group to the mean effect of all subsequent binge/purge frequency groups (e.g., Group 1 vs. average of Group 2, Group 3, Group 4; Group 2 vs. average of Group 3, Group 4; Group 3 vs. Group 4) on clinical characteristics. Helmert contrasts were selected so that we could test how the SEM Tree group most consistent with AN‐R differed from all groups with higher binge/purge frequency and so that we could test whether higher binge/purge frequency groups differed from one another. We used partial eta squared to indicate overall effect sizes and Cohen's *d* for contrast effect sizes and included 95% confidence intervals for effect sizes. Chi‐square analyses were used to compare psychiatric comorbidity, suicide history, and percent of clinical AN‐BP diagnoses. Pairwise z‐comparisons were used to examine differences between groups for significant tests, and Cramer's *V* effect sizes were calculated for all Chi‐Square tests.

## 3 Results

### 3.1 SEM Tree

The SEM Tree identified three cutpoints on past‐month binge+purge frequency, which resulted in four groups (Figure 1): Group 1 (AN‐BP0; past month binge+purge frequency < 0.5; *n* = 396); Group 2 (AN‐BP1; past month binge+purge frequency ≥ 0.5 and < 3.5; *n* = 101); Group 3 (AN‐BP2; past month binge+purge frequency ≥ 3.5 and < 16; *n* = 130); and Group 4 (AN‐BP3; past month binge+purge frequency ≥ 16; *n* = 98).

Because it was not possible for an individual to have 0.5 or 3.5 episodes in the past month, we round these numbers to the nearest whole number from this point forward to facilitate the practical implications of these findings. As such, we consider the first split to be between people with 0 vs. 1 binge or purge episode. The second split was between people with 1–3 vs. 4 binge or purge episodes. The third split was between people with 4–15 vs. ≥ 16 binge or purge episodes. We consider AN‐BP0 (AN‐R‐like) to include individuals with 0 binge + purge episodes in the past month, AN‐BP1 to include individuals with 1–3 binge + purge episodes in the past month, AN‐BP2 to include individuals with 4–15 binge + purge episodes in the past month, and AN‐BP3 to include individuals with 16 or more binge + purge episodes in the past month.

### 3.2 Group Comparisons

Chi‐square tests and ANOVAs showed SEM Tree groups differed on age, clinical diagnosis, presence of current mood, alcohol use, and substance use disorders, suicide risk, history of suicide attempts, EDE‐Q Global and all subscales, 5 of the 6 EPSI subscales, impulsivity, punishment sensitivity measured by the SPSRQ, and clinical impairment (Table 1). SEM Tree groups did not differ on the presence of current anxiety disorders, BMI, or punishment sensitivity measured by the BIS.

**TABLE 1:** Clinical characteristics compared among the structural equation model tree‐derived groups in Model 1 (total binge/purge frequency).

	AN‐BP0	AN‐BP1	AN‐BP2	AN‐BP3			
	BP < 0.5	3.5 < BP ≥ 0.5	16 < BP ≥ 3.5	BP ≥ 16			
	*n* = 396	*n* = 101	*n* = 130	*n* = 98			
	*n* (%)	*n* (%)	*n* (%)	*n* (%)	χ ^2^	*p*	Cramer's *V*
Clinical diagnosis (% AN‐BP)	33 (8%)^b,c,d^	20 (20%)^a,c,d^	65 (50%)^a,b,d^	77 (78%)^a,b,c^	238.54	< 0.001	0.57
Current anxiety disorder	198 (51%)	55 (57%)	74 (59%)	60 (63%)	5.52	0.14	0.10
Current mood disorder	164 (42%)^c,d^	48 (49%)^d^	76 (60%)^a^	65 (69%)^a,b^	28.19	< 0.001	0.20
Current alcohol use disorder	16 (4%)^d^	2 (2%)^d^	9 (7%)	15 (16%)^a,b^	21.95	< 0.001	0.18
Current substance use disorder	21 (5%) ^c^	9 (9%)	17 (13%) ^a^	12 (13%)	11.00	0.01	0.13
Suicidality score (% High Risk)	28 (12%)^b^	16 (28%)^a^	15 (19%)	14 (26%)	18.29	0.03	0.12
History of ≥ 1 suicide attempt	20 (9%)^c^	6 (10%)	21 (27%)^a^	10 (19%)	24.34	< 0.001	0.17

	*M* (SD)	*M* (SD)	*M* (SD)	*M* (SD)	*F*	*p*	η ^2^	η ^2^ 95% CI
Age*	19.1 (7.0)	18.3 (5.5)	19.9 (7.3)	22.2 (9.6)	4.50	0.01	0.02	0.01–0.05
BMI	17.8 (2.0)	17.7 (1.9)	18.1 (1.9)	18.4 (1.7)	3.07	0.03	0.01	0.00–0.03
Past‐month binge frequency^§^	0.0 (0.0)	1.1 (1.0)	3.7 (3.8)	13.5 (16.0)	—	—	—	—
Past‐month purge frequency^§^	0.0 (0.0)	0.8 (1.0)	4.7 (4.3)	26.5 (19.2)	—	—	—	—
EDE‐Q global*	2.9 (1.7)	3.6 (1.3)	4.2 (1.3)	4.5 (1.1)	49.97	< 0.001	0.15	0.10–0.19
EDE‐Q Restraint*	2.5 (2.0)	3.0 (1.9)	3.9 (1.7)	4.2 (1.5)	36.62	< 0.001	0.12	0.07–0.16
EDE‐Q eating concern*	2.4 (1.5)	3.1 (1.3)	3.6 (1.2)	4.0 (1.0)	58.34	< 0.001	0.17	0.12–0.22
EDE‐Q weight concern*	3.1 (2.0)	3.8 (1.6)	4.4 (1.4)	4.7 (1.4)	37.58	< 0.001	0.12	0.08–0.16
EDE‐Q Shape Concern*	3.8 (1.9)	4.4 (1.5)	4.9 (1.3)	5.2 (1.2)	32.71	< 0.001	0.10	0.06–0.14
EPSI body dissatisfaction*	17.4 (7.4)	18.1 (6.7)	20.7 (5.5)	21.6 (6.1)	8.36	< 0.001	0.06	0.02–0.11
EPSI binge eating*, ^‡^	3.6 (4.1)	6.0 (5.1)	8.4 (6.6)	10.7 (7.8)	32.30	< 0.001	0.20	0.13–0.26
EPSI cognitive restraint*	7.5 (4.1)	8.6 (3.3)	9.1 (2.8)	8.6 (3.4)	4.40	0.01	0.03	0.00–0.07
EPSI Purging*, ^‡^	1.1 (2.7)	2.7 (3.3)	4.7 (4.2)	5.8 (4.5)	37.78	< 0.001	0.23	0.15–0.29
EPSI Restricting	14.6 (6.5)	16.2 (5.2)	16.7 (5.5)	16.7 (6.1)	3.44	0.02	0.03	0.00–0.06
EPSI Excessive Exercise	7.8 (7.0)	9.7 (6.5)	9.7 (6.5)	9.3 (7.1)	2.44	0.06	0.02	0.00–0.05
TCI‐NS	16.6 (6.7)	16.6 (5.9)	17.5 (6.9)	20.5 (7.4)	2.74	0.04	0.03	0.00–0.08
SPSRQ‐SP*	9.5 (3.6)	10.0 (3.4)	10.7 (3.0)	10.3 (2.7)	3.12	0.03	0.02	0.00–0.04
BIS	24.2 (3.4)	24.1 (3.3)	24.6 (2.8)	24.2 (2.9)	0.64	0.59	< 0.01	0.00–0.01
CIA*	28.2 (13.0)	31.4 (12.4)	35.2 (10.3)	36.5 (7.7)	14.59	< 0.001	0.08	0.03–0.13

*Note:* For pairwise z‐comparisons of Chi‐Square tests, a, differs significantly from AN‐R; b, differs significantly from AN‐BP1; c, differs significantly from AN‐BP2; d, differs significantly from AN‐BP3. *n* differs for current comorbidities, suicidality score, history of suicide attempts, BMI, EPSI, TCI‐NS, SPSRQ‐SP, BIS, and CIA as a result of missing data.

Abbreviations: AN‐BP = anorexia nervosa, binge‐purge subtype; BMI = body mass index (kg/m^2^); BP=Past‐month binge/purge frequency; CIA = Clinical Impairment Assessment; EDE‐Q = Eating Disorders Examination Questionnaire; EPSI = Eating Pathology Symptoms Inventory; SPSRQ‐SP=Sensitivity to Punishment Sensitivity to Reward Questionnaire‐Sensitivity to Punishment Subscale; BIS=Behavioral Inhibition System Scale; TCI‐NS = Temperament and Character Inventory‐Novelty Seeking Subscale.

* Indicates unequal variances across groups.

** Indicates that although unequal variance across groups, Welch correction could not be applied due to one group having zero variance.

§ Mean values and standard deviations for past‐month binge and purge frequency based on the EDE‐Q are presented, but were not statistically compared given that groups were derived on these variables.

‡ EPSI binge eating and purging scores provide severity scores for binge eating and purging, respectively, not provide past‐month frequencies of binge eating and purging.

Post hoc comparisons of Chi‐square tests suggest that the percentage of participants with a clinical AN‐BP diagnosis increased as the level of binge + purge frequency increased. Presence of mood disorders was greater in AN‐BP2 and AN‐BP3 relative to AN‐BP0 and in AN‐BP3 relative to AN‐BP1. Presence of alcohol use disorder was greater in AN‐BP3 compared to AN‐R and AN‐BP1. Presence of substance use disorder and history of at least one suicide attempt were greater in AN‐BP2 relative to AN‐BP0. The percentage of participants classed as high suicide risk was higher in AN‐BP1 relative to AN‐BP0.

Post hoc comparisons of Chi‐square tests and planned contrasts (Table 2) indicated that AN‐BP0 differed from higher frequency groups on 14/23 clinical characteristics, with *mean* effect size = 1.42. AN‐BP1 differed from higher frequency groups on 12/23 clinical characteristics, with *mean* effect size = 0.69. AN‐BP2 differed from AN‐BP3 on 2/23 clinical characteristics, with *mean* effect size = 0.14.

**TABLE 2:** Model 1 structural equation model tree‐derived groups‘ contrast results.

	AN‐BP0 vs. (AN‐BP1, AN‐BP2, AN‐BP3)				AN‐BP1 vs. (AN‐BP2, AN‐BP3)				AN‐BP2 vs. AN‐BP3			
	*t* (df)	*p*	*d*	*d* 95% CI	*t* (df)	*p*	*d*	*d* 95% CI	*t* (df)	*p*	*d*	*d* 95% CI
Age	3.05 (530.26)	0.06	0.42	−0.02‐0.86	5.54 (263.78)	< 0.001	0.76	0.29–1.23	2.25 (177.01)	0.05	0.31	0.05–0.57
BMI	1.91 (719)	0.06	0.43	−0.01‐0.87	2.19 (719)	0.03	0.53	0.06–1.00	0.94 (719)	0.35	0.13	−0.14‐0.39
EDE‐Q global*	10.49 (695.46)	< 0.001	2.29	1.83–2.74	4.82 (171.66)	< 0.001	0.99	0.52–1.47	2.07 (221.96)	0.04	0.22	−0.05‐0.48
EDE‐Q restraint*	8.0.61 (692.50)	< 0.001	1.90	1.45–2.35	4.68 (169.53)	< 0.001	1.08	0.61–1.56	1.40 (222.63)	0.17	0.16	−0.10‐0.42
EDE‐Q eating concern*	11.60 (693.56)	< 0.001	2.54	2.08–3.00	4.33 (167.76)	< 0.001	0.92	0.45–1.39	2.83 (220.70)	0.01	0.30	0.04–0.56
EDE‐Q weight concern*	9.20 (690.68)	< 0.001	2.02	1.56–2.47	4.13 (168.56)	< 0.001	0.88	0.40–1.35	1.57 (213.62)	0.12	0.17	−0.10‐0.43
EDE‐Q shape concern*	8.77 (685.22)	< 0.001	1.91	1.45–2.36	3.52 (170.89)	< 0.001	0.70	0.23–1.17	1.73 (215.77)	0.09	0.17	−0.09‐0.44
EPSI body dissatisfaction*	4.00 (354.59)	< 0.001	1.22	0.61–1.83	2.91 (86.09)	0.01	0.91	0.26–1.55	0.90 (106.12)	0.37	0.14	−0.21‐0.48
EPSI binge eating*	8.42 (240.32)	< 0.001	2.66	2.03–3.29	3.65 (127.86)	< 0.001	1.29	0.64–1.94	1.80 (101.88)	0.08	0.43	0.08–0.78
EPSI cognitive restraint*	3.40 (356.42)	< 0.001	1.03	0.43–1.64	0.48 (92.64)	0.63	0.14	−0.50‐0.78	−0.89 (98.99)	0.37	−0.14	−0.49‐0.21
EPSI purging*	9.15 (274.32)	< 0.001	2.86	2.22–3.49	4.25 (124.70)	< 0.001	1.49	0.84–2.14	1.34 (110.00)	0.18	0.31	−0.04‐0.65
EPSI restricting	3.11 (388)	0.002	0.95	0.35–1.56	0.47 (388)	0.64	0.15	−0.49‐0.80	−0.05 (388)	0.96	−0.01	−0.36‐0.34
EPSI excessive exercise	2.63 (387)	0.01	0.81	0.20–1.41	−0.17 (387)	0.87	−0.06	−0.70‐0.59	−0.35 (387)	0.73	−0.06	−0.41‐0.29
TCI‐NS	1.80 (242)	0.07	0.70	−0.07‐1.47	1.78 (242)	0.09	0.71	−0.12‐1.53	1.66 (242)	0.10	0.44	−0.05‐0.92
SPSRQ‐SP*	2.50 (460.43)	0.01	0.68	0.13–1.22	1.09 (114.14)	0.28	0.31	−0.26‐0.88	−0.90 (141.12)	0.37	−0.12	−0.45‐0.20
BIS	0.35 (620)	0.73	0.09	−0.39‐0.72	0.83 (620)	0.41	0.22	−0.39‐0.72	−0.94 (620)	0.35	−0.14	−0.42‐0.15
CIA*	5.24 (344.24)	< 0.001	1.58	0.97–2.19	2.40 (74.96)	0.02	0.76	0.12–1.40	0.83 (130.89)	0.41	0.11	−0.24‐0.45

Abbreviations: BIS=Behavioral Inhibition System Scale; BMI = body mass index (kg/m^2^); CIA = Clinical Impairment Assessment; EDE‐Q = Eating Disorders Examination Questionnaire; EPSI = Eating Pathology Symptoms Inventory; SPSRQ‐SP=Sensitivity to Punishment Sensitivity to Reward Questionnaire‐Sensitivity to Punishment Subscale; TCI‐NS = Temperament and Character Inventory‐Novelty‐Seeking Subscale.

* Indicates significant heterogeneity of variance and thus Welch‐corrected contrasts are reported.

## 4 Discussion

The present study used SEM trees to empirically derive the frequency of binge eating and purging that differentiates AN subtypes and compared SEM tree‐derived groups on clinically relevant variables. Three splits were identified at increasing frequencies of binge eating and purging, creating four AN subgroups. The SEM tree‐derived AN‐R and AN‐BP groups differed in clinical characteristics consistent with those that have differentiated clinical AN‐R and AN‐BP diagnoses in prior research.

The first SEM tree cutpoint created a subgroup (AN‐BP0) with no binge eating or purging in the past month, similar to the clinical AN‐R subtype, and left three other subgroups who experienced nonzero binge eating and/or purging, similar to the clinical AN‐BP subtype. In line with the hypotheses, the AN‐R‐like subgroup had lower ED symptoms, psychiatric comorbidity, suicidality, impulsivity, punishment sensitivity measured by the SPSRQ, and clinical impairment than AN‐BP‐like groups. These results coincide with findings illustrating differences in clinical characteristics between AN subtypes (Bulik et al. 2008; Garner, Garner, and Rosen 1993; Glashouwer et al. 2014; Mond et al. 2005; Waxman 2009; Reas and Rø 2018) and taxometric research suggesting qualitative distinctions between AN‐R and EDs characterized by binge eating (Gleaves et al. 2000; Williamson et al. 2002; Wonderlich et al. 2007). Overall, results suggest that one binge‐eating and/or purging episode in the past month is the point at which AN‐BP differs from AN‐R. This cutoff is consistent with what has been used in some prior work that distinguishes between AN‐R and AN‐BP (Garner, Garner, and Rosen 1993; Mond et al. 2005). Clinically, distinguishing individuals with any binge eating and/or purging behaviors, regardless of frequency, make sense given that binge eating and purging may require targeted intervention whenever present, regardless of frequency.

Although the distinction between the AN‐R‐like group and the AN‐BP‐like groups was clear (i.e., largest effect sizes, most group differences), distinction among the three AN‐BP‐like subgroups was less strong and less consistent. Individuals in AN‐BP2 and AN‐BP3 had more binge/purge episodes (4–15 and ≥ 16, respectively) compared to people in AN‐BP1 (1–3 episodes) and had higher symptom load than people with AN‐BP1 on 12/23 characteristics, with an average effect size roughly half of the average effect size for differentiating the AN‐R‐like group from AN‐BP‐like groups. Individuals with AN‐BP3 had more binge/purge episodes than people with AN‐BP2 and had higher symptom load than AN‐BP2 on only 2/23 characteristics with small effect sizes. These results beg the question of whether having multiple AN‐BP subtypes, defined by increasing frequency of binge eating and purging, is empirically justified and clinically useful. On the one hand, a dimensional conceptualization of binge/purge frequency in AN may increase diagnostic precision and help personalize treatment to address specific symptoms (Forbush et al. 2018). For example, patients with few binge eating and/or purging episodes may require less focus on changing these behaviors in treatment while patients with many episodes may require significant focus on these behaviors. On the other hand, a dimensional approach to AN subtype differentiation based on binge/purge frequency is similar to the *DSM‐5‐TR*'s severity specifiers for bulimia nervosa and binge‐eating disorder, which are defined by increasing frequencies of ED behaviors (compensatory behaviors in bulimia nervosa, binge eating in binge‐eating disorder). Research on the utility of the *DSM‐5‐TR*'s severity definitions has yielded minimal to mixed support (Billman Miller et al. 2024; Forrest, Jacobucci, and Grilo 2022; Gianini et al. 2017; Gorrell et al. 2019; Grilo et al. 2009). Given that increasing frequencies of compensatory behaviors and binge‐eating episodes do not reliably signal severity differences in other EDs (Billman Miller et al. 2024; Forrest, Jacobucci, and Grilo 2022; Ortiz et al. 2021) and clinical severity distinctions in our sample are less pronounced at higher levels of binge/purge frequency, it is perhaps unsurprising that increasing frequency of binge eating/purging did not reliably signal meaningful differences within AN‐BP‐like groups in the current study. Taken together, we believe that the present results do not support adopting a dimensional approach for AN‐BP subtypes. Rather, results support a zero vs. nonzero conceptualization of binge eating and purging when determining AN subtypes. Individuals who report no binge eating or purging should be considered as having AN‐R, while individuals who report any binge eating or purging should be considered as having AN‐BP. Notably, this zero vs. nonzero conceptualization is at odds with the *DSM‐5‐TR*'s definition of “recurrent” binge eating or purging and suggest that the future iterations of the *DSM* could consider removing “recurrent” from AN subtype definitions.

Strengths of this study included a large clinical sample comprising both adolescent and adult participants, suggesting findings may generalize across different age groups, and the use of machine learning to empirically derive a cutpoint for binge/purge frequency. Further research is needed on this topic, though, given several limitations that limit generalizability. First, there was minimal ethnoracial and gender diversity within the study sample. Second, self‐induced vomiting and laxative use were the only types of purging measured by the EDE‐Q. While self‐induced vomiting and laxative use are the most common methods of purging (Edler, Haedt, and Keel 2007; Mond et al. 2006; Murakami et al. 2002), results may not apply to individuals who purge through diuretic use or other means. Third, the study sample solely comprised ED treatment‐seeking individuals and inter‐rater reliability data were not available for clinical interviews used for diagnosis. Treatment‐seeking samples may differ from those that do not seek treatment (Forrest, Jacobucci, and Grilo 2022). As such, results might not generalize to nontreatment‐seeking individuals. Further, as the sample was drawn from a partial hospitalization program, versus from lower levels of care such as outpatient and IOP, findings may have been biased toward higher levels of binge eating/purging severity. Future research is necessary to replicate these findings among those in lower‐intensity treatment settings and in nontreatment‐seeking samples. Fourth, binge‐eating and purging frequencies were calculated based on the EDE‐Q, which provides past‐month frequency of these behaviors and relies on participant recall and reporting of episodes. *DSM‐5‐TR* specifies a 3‐month timeframe for many ED behaviors, and it is unknown how our results may generalize to assessments over longer timeframes. Fifth, some work suggests that a subjective sense of loss of control is a more central characteristic of binge eating than episode size (Latner et al. 2007), which could indicate that individuals with AN and subjective binge‐eating episodes are more similar to those with AN‐BP than those with AN‐R. However, the EDE‐Q does not include a metric of subjective binge‐eating episodes, so the present study was not able to shed light on how subjective binge‐eating episodes may influence our findings. Finally, limited variables were available from which to create the outcome model and compare subgroups on outcomes. Since AN frequently involves significant medical morbidity (Mehler and Brown 2015; Westmoreland, Krantz, and Mehler 2016), it would be beneficial for future studies to include physiological variables as well as self‐reported symptoms when constructing the AN diagnosis latent variable.

An additional question for future research to consider related to AN subtypes is whether binge eating and purging are synonymous when differentiating AN‐R and AN‐BP. The *DSM‐5‐TR* assigns the AN‐BP subtype to those engaging in *either* binge eating or purging. In support of this umbrella conceptualization, some work finds few differences among people with AN who binge but do not purge (“AN‐B”), purge but do not binge (“AN‐P”), and both binge and purge (“AN‐BP”) (Peterson et al. 2016). However, other research suggests nonequivalence between binge eating and purging in people with AN. For example, Klump et al. (2000) found that individuals with AN‐P have higher harm avoidance than people with AN‐R, AN‐B, and AN‐BP. Other research suggests that the presence of purging—regardless of the presence of binge eating—across ED diagnoses generally is associated with more severe psychiatric symptoms (Favaro and Santonastaso 1996). Future research to reconcile this mixed literature would be helpful.

In summary, the current study empirically identified the frequency of binge eating and/or purging that best differentiates AN‐R from AN‐BP. Results identified four AN subgroups: one AN‐R‐like group, where individuals reported no past‐month binge eating or purging, and three AN‐BP‐like groups, which were defined by increasing frequencies of binge eating and purging. The AN‐R‐like group most strongly and consistently differed from the AN‐BP‐like groups, while differences among the AN‐BP‐like groups were less consistent and had smaller effect sizes, suggesting dimensionality in AN severity at lower levels of binge/purge frequency that is obscured at higher levels of binge/purge frequency. Future iterations of the *DSM* should consider removing language about “recurrent” binge eating and/or purging for distinguishing AN subtypes, and instead refer to a specific cutpoint. The present results suggest that such a cutpoint is one binge/purge episode, though this threshold needs replication, particularly in samples with more diversity (e.g., in demographic characteristics, level of care, and treatment‐seeking vs. nontreatment‐seeking) and using the *DSM*'s 3‐month timeframe. Regardless of whether future research replicates the binge/purge cutpoint identified here, we urge the field to implement standardized definitions of AN subtypes across research and clinical sites. This standardization is critical for advancing our understanding of AN.

## Author Contributions

**Sophie R. Abber:** conceptualization, formal analysis, methodology, visualization, writing – original draft, writing – review and editing. **Devon Peterkin:** writing – original draft, writing – review and editing. **Carina S. Brown:** writing – review and editing. **Thomas E. Joiner:** writing – review and editing. **Christina E. Wierenga:** conceptualization, funding acquisition, methodology, project administration, | resources, supervision, writing – review and editing. **Lauren N. Forrest:** conceptualization, formal analysis, methodology, supervision, writing – review and editing.

## Ethics Statement

The authors assert that all procedures contributing to this work comply with the ethical standards of the relevant national and institutional committees on human experimentation and with the Helsinki Declaration of 1975, as revised in 2008. The current study was approved by the University of California, San Diego's Institutional Review Board (180055).

## Conflicts of Interest

The authors declare no conflicts of interest.

## Supporting information


**Data S1.** AN Cutoff SEM Trees_code for pub.

## Data Availability

The data that support the findings of this study are available from the corresponding author upon reasonable request.
