# Supplementary material for: Empirically Determining Binge/Purge Frequency Thresholds for Differentiating Anorexia Nervosa‐Restricting Subtype vs. Binge–Purge Subtype
Source: Int J Eat Disord. 2025 Feb 6;58(5):868–77. doi: 10.1002/eat.24391 (PMC12067515; doi:10.1002/eat.24391)
Supplement: Supplementary file 1 — Data S1. AN Cutoff SEM Trees_code for pub. [file EAT-58-868-s001.docx]

#---------------AN Cutoff SEM Trees---------------#

#0. load packages

library(foreign)

library(haven)

library(OpenMx)

library(lavaan)

library(devtools)

devtools::install_github("brandmaier/semtree", force = TRUE)

#read in AN_fiml data; this dataframe includes only five variables: the three indicators of the outcome model (AN criterion A, B, and C) and the covariate (binge frequency and purge frequency)

AN_observed_Model1=colnames(AN3_model1[,c(1,2,3)])

fitFunction <- mxFitFunctionML(rowDiagnostics=TRUE)

AN_latent_Model1=c("f1")

AN_cfa_Model1<-mxModel("Model", type="RAM",

manifestVars=c(AN_observed_Model1),

latentVars=c("f1"),

#factor loadings

mxPath(from="f1", to=c("CritA","CritB","CritC"),

free=c(F,F,F),values=c(1,1,1),labels=c("l1","l2","l3")),

#manifest means

mxPath(from = 'one', to = AN_observed_Model1,values=2,labels=paste(paste("m"), 1:3,sep="")),

#residual variances

mxPath(from=AN_observed_Model1, arrows=2,labels=paste(paste("resid"), 1:3,sep=""),lbound=0),

#latent mean

#mxPath(from = 'one', to = "f1",values=1,free=F),

#latent variance

mxPath(from=AN_latent_Model1, arrows=2,free=F,values=1,labels=c("v1")),

fitFunction,

#data

mxData(AN3_model1[c(1,2,3)],type="raw")

)

AN_cfa.out_Model1 = mxRun(AN_cfa_Model1)

summary(AN_cfa.out_Model1)

AN3_model1 <- as.data.frame(AN3_model1)

#OpenMx tree

my.control = semtree.control(method="fair", seed=1, min.N=40, missing = "party")

AN_tree_Model1 <- semtree(model = AN_cfa.out_Model1, data = AN3_model1, control = my.control)

pdf("AN_tree_Model1.pdf")

plot(AN_tree_Model1)

dev.off()

beep(sound = 3)
